# Supplementary material for: The genome sequence of an ichneumonid wasp, Rhorus exstirpatorius (Gravenhorst, 1829)
Source: Wellcome Open Res. 2024 Aug 7;9:426. [Version 1] doi: 10.12688/wellcomeopenres.22877.1 (PMC11530747; doi:10.12688/wellcomeopenres.22877.1)
Supplement: Peer review history

# Reviewer report: Reviewer response for version 1

**DOI:** 10.21956/wellcomeopenres.25192.r103097

**Published:** 2024-11-01

**Authors:** Sara E Miller

**Affiliations:** 1 Department of Biology, University of Missouri St. Louis, St Louis, Missouri, USA

The authors present a new high quality nuclear and mitochondrial genome assembly for the ichneumonid wasp *Rhorus exstirpatorius *(Hymenoptera: Ichneumonidae). Parasitoid of sawflies.

 The genome was constructed for single male using Pacific Biosciences HiFi circular consensus DNA libraries, sequenced on the Pacific Bioscience Sequel IIe, and scaffolded with Hi-C data. Raw data and assembly methods are available. The final assembly is 420.3 Mb in length with a scaffold N50 of 42.4 Mb. The genome assembly has 94.7% coverage of BUSCO genes, however there is no annotation currently available for this assembly. 

 I appreciate the inclusion of a photograph of the specimen used to construct the genome assembly in Figure 1, however, the specimen is covered in frost, has the head unattached, and does not include a scale bar. This makes it challenging for the reader to make out features of this species or appreciate how this species fits into the substantial variation in body size and shape of this genus that is mentioned in the introduction. If a second specimen is available, I suggest adding an additional photograph that more clearly shows organismal features.

 Ichneumonid wasps are one of the most specious groups of Hymenoptera, but are underrepresented in current Hymenoptera genome assemblies. This new, high-quality assembly of *R. exstirpatorius,* increases our understanding of this understudied group.

Are sufficient details of methods and materials provided to allow replication by others?

Yes

Is the rationale for creating the dataset(s) clearly described?

Yes

Are the datasets clearly presented in a useable and accessible format?

Yes

Are the protocols appropriate and is the work technically sound?

Yes

Reviewer Expertise:

Social insect genetics, and genomics, evolutionary ecology.

I confirm that I have read this submission and believe that I have an appropriate level of expertise to confirm that it is of an acceptable scientific standard.

# Reviewer report: Reviewer response for version 1

**DOI:** 10.21956/wellcomeopenres.25192.r98312

**Published:** 2024-09-25

**Authors:** Yi Wang

**Affiliations:** 1 Southwest University, Chongqing, Chongqing, China

This paper focuses on the genome sequencing of *Rhorus exstirpatorius* (an ichneumonid wasp), presenting a comprehensive workflow from sample collection, DNA/RNA extraction, to genome assembly and evaluation. The study utilizes high-quality HiFi long-read sequencing and HiC data, resulting in a high-coverage, high-quality genome assembly.  The article provides detailed descriptions of the genome assembly methods, evaluation metrics, and data archiving paths, offering a valuable reference for future research. This study holds significant scientific importance and offers considerable data value, especially as it fills a gap in the genomic research of ichneumonid wasps. I believe the study is worthy of Indexing, but there are a few details that need further refinement and clarification.

**Areas for Improvement:**

**1. Insufficient Background in the Introduction:** Although the article briefly outlines the taxonomic position and biological characteristics of *Rhorus exstirpatorius*, the discussion regarding its ecological, economic, or evolutionary significance is rather limited.  It is recommended to expand on the species' role in the ecosystem, or on the potential applications of its genome sequencing in evolutionary research or biological control.  This would help to further highlight the importance and practical relevance of the study.

**2. Insufficient Genome Functional Annotation:** The article primarily focuses on the structural aspects of the genome (e.g., N50 value, total length, chromosome-level assembly, etc.), but provides little information on the functional aspects of the genome (e.g., gene count, gene function annotations, metabolic pathways).  While the paper mentions the use of RNA-Seq data for genome annotation and its display via the Ensembl pipeline, no specific functional annotation results are provided in this draft.  It is recommended to include some preliminary functional annotation information, such as simple gene count statistics or the identification of important genes, to enrich the paper's content.

**3. Further Refinement of Species Identification Methods: **Although the article mentions the use of COI barcoding for species identification, it does not provide detailed results of this process.  It is recommended to include the barcode sequence alignment results and specific identification parameters (e.g., percentage similarity, choice of reference database, etc.), which would enhance the transparency and accuracy of the species identification process.

Are sufficient details of methods and materials provided to allow replication by others?

Yes

Is the rationale for creating the dataset(s) clearly described?

Yes

Are the datasets clearly presented in a useable and accessible format?

Yes

Are the protocols appropriate and is the work technically sound?

Yes

Reviewer Expertise:

Genome; Bioinformatics

I confirm that I have read this submission and believe that I have an appropriate level of expertise to confirm that it is of an acceptable scientific standard.
